# Interpretable machine learning uncovers core seminal microbial signatures in idiopathic oligoasthenospermia

**DOI:** 10.3389/fcimb.2026.1923816

**Published:** 2026-09-16

**Authors:** Shikuan Lu, Yipeng Zhao, Yuxin Zhao, Huangtang Dong, Chunxu Qu, Weicai Zhong, Peihai Zhang, Ziyang Ma, Pengfei Zhang

**Affiliations:** 1 Hospital of Chengdu University of Traditional Chinese Medicine, Chengdu, China; 2 Institute of Andrology, Chengdu University of Traditional Chinese Medicine, Chengdu, China; 3 School of Intelligent Medicine, Chengdu University of Traditional Chinese Medicine, Chengdu, China

**Keywords:** 16S rRNA sequencing, artificial intelligence, idiopathic oligoasthenospermia, machine learning, precision medicine, semen microbiota

## Abstract

**Background:**

Previous studies have confirmed that changes in semen microbiota are closely related to male oligoasthenospermia(OA). However, current research only qualitatively describes the differences in the microbial community, fails to screen out core markers with independent discriminatory value, and also fails to quantitatively evaluate the diagnostic efficacy of the microbial community, resulting in insufficient research on the diagnostic value of the semen microbiota in oligoasthenospermia.

**Methods:**

A total of 40 untreated patients with idiopathic oligoasthenospermia (IOA) and 30 fertile control (FC) were recruited for this study. The semen samples were sequenced using 16S rRNA sequencing technology to assess the differences in microbial diversity and abundance. Subsequently, three machine learning algorithms were employed to further identify the core microorganisms and to further evaluate the diagnostic performance. Then, the SHapley Additive exPlanations(SHAP)analysis method was used to explain the contribution of these core microbiota to the disease. Additionally, the Phylogenetic Investigation of Communities by Reconstruction of Unobserved States 2 (PICRUSt2) algorithm was used to predict the functions of the core microorganisms.

**Results:**

The analysis of the microbial community in semen revealed that there were differences in the internal composition structure of the semen microbiota between the IOA group and the FC group. Machine learning algorithms identified a total of 5 core bacterial genera. The area under the curve (AUC) of this model was 0.773, with a 95% confidence interval (CI) of 0.661–0.885, indicating that the model has strong discriminatory power. SHAP analysis further revealed the direction of the association between the abundance of the microbiota and disease prediction. Additionally, KEGG pathway prediction revealed that the 5 core genera were predominantly enriched in carbohydrate and nucleic acid metabolism pathways, most notably glycolysis/gluconeogenesis (*P* = 6.67 × 10^-3^).

**Conclusion:**

This study employed a variety of machine learning algorithms to conduct a systematic characterization study on the semen microbiota of patients with idiopathic oligoasthenospermia. This method overcomes the limitations of traditional microbiota analysis and can This approach overcame the limitations of traditional microbiota analysis and constructed a risk prediction model based on core bacterial genera. It preliminarily explored the potential of the semen microbiota as a non-invasive screening candidate marker for IOA, providing a reference for subsequent research on non-invasive diagnostic markers and mechanisms., providing a basis for non-invasive precise diagnosis of the disease and the analysis of the pathogenic mechanism of the microbiome.

## 1 Introduction

Infertility is a global issue. The World Health Organization estimates that approximately one in six people of reproductive age worldwide experience infertility during their lifetime, with about half of these cases attributed to male factors (World Health Organization, 2023). Idiopathic oligoasthenospermia (IOA) is one of the most common types of male infertility in clinical practice. Patients only present with decreased sperm concentration and reduced sperm motility. If no clear organic lesions of the reproductive system, endocrine disorders, or known pathogenic microbial infections are identified as the causal factors, the cause is obscure and the pathogenesis has not been fully elucidated (Zhang J et al., 2024). Currently, there are no specific diagnostic indicators or precise intervention methods for this disease in clinical practice. The treatment plans are relatively simple, and the overall treatment effect is limited. In the era of precision medicine, the screening of new non-invasive biomarkers and the development of more accurate diagnostic tools are particularly crucial for the early clinical identification of idiopathic oligoasthenospermia (IOA).

Previous studies have confirmed (Chen et al., 2023; Chatzokou et al., 2025; Zuber et al., 2023) that the stability of the reproductive tract microecology is closely related to the normal reproductive function of men. The semen is colonized by a structurally stable symbiotic microbial community, which maintains the local microenvironment balance and participates in regulating the process of spermogenesis and maturation, playing a crucial protective role in sperm morphology and motility. Once the balance of the microbiota is disrupted, harmful bacteria multiply abnormally and the abundance of beneficial bacteria decreases, which can trigger local chronic inflammation, oxidative stress disorders, and sperm energy metabolism disorders, interfering with the spermatogenesis process, and ultimately leading to a decline in semen quality and promoting the occurrence and development of idiopathic oligoasthenospermia (Farahani et al., 2021; Davies et al., 2023; Magoutas et al., 2025). However, most of the existing studies have adopted traditional differential analysis methods. Although they have initially confirmed the association between dysbiosis of the microbiota and oligoasthenospermia, it is difficult to precisely screen out the core microbiota from high-dimensional data (Santos-Júnior et al., 2024), and it is also impossible to quantify the contribution weight of the microbiota, clarify its direction of action and the regulatory rules of abundance dependence, making it difficult to elucidate the intrinsic mechanism of the microbiota’s involvement in disease occurrence and limiting the clinical reference value.

In recent years, interpretable machine learning methods have gradually been applied in microbiomics research, providing new tools to address the shortcomings of the traditional approaches. the Least Absolute Shrinkage and Selection Operator (LASSO) regression, by applying L1 regularization, can achieve efficient dimensionality reduction of high-dimensional microbiome data, eliminating redundant and noisy species while retaining the core features highly related to the outcome (Fei et al., 2024). Compared to a single algorithm, by combining multiple algorithms such as Random Forest(RF), Support Vector Machine - Recursive Feature Elimination (SVM-RFE) for parallel screening and locking the final feature set through intersection, the robustness and reproducibility of feature selection can be significantly improved (Chen et al., 2026). On this basis, SHAP explainability analysis can further quantify the independent influence degree of each bacterial genus on the occurrence of the disease, intuitively distinguish the protective and pathogenic effects of the bacterial groups, clarify the differential biological effects of the bacterial groups at different abundance levels, and effectively make up for the shortcomings of traditional bacterial group analysis (Novielli et al., 2025).

In this context, this study analyzed the microbial structure of the semen of IOA patients based on 16S rRNA sequencing. An interpretable machine learning model was used to screen the core microbiota, and combined with SHAP analysis and functional prediction(**Figure 1**), the aim was to identify stable microbial markers and elucidate their potential mechanisms in the occurrence and development of oligoasthenospermia.

**Figure 1:**
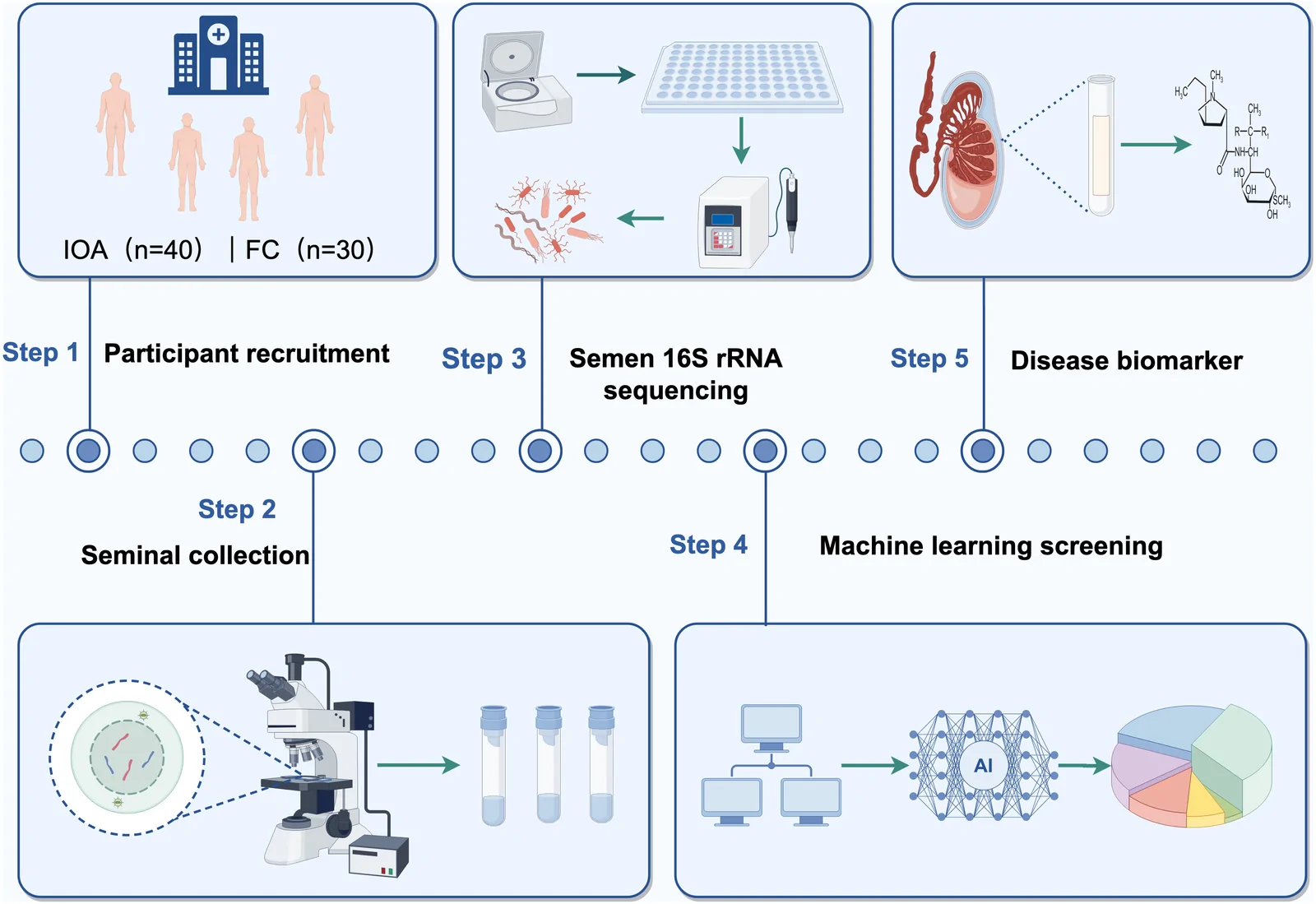
The workflow for sample collection, data processing, analysis, and the construction of machine learning models. Created with Figdraw, copyright code: RUPSWa2933.

## 2 Materials and methods

### 2.1 Participant recruitment

The subjects included 40 outpatients from Chengdu University of Traditional Chinese Medicine Affiliated Hospital and 30 healthy subjects from July 2024 to March 2025.

Healthy male subjects must meet the following conditions: Their partners have given birth or been pregnant within the past year. The results of two semen tests (with an interval of 2 to 4 weeks) are both normal (sperm concentration ≥ 15×10^6^/mL, forward-moving sperm ≥ 32%, normal morphology ≥ 4%).An IOA patient must first meet the diagnostic criteria for oligoasthenospermia as stipulated in the fifth edition of the “Laboratory Manual for Human Semen Examination and Processing” by the World Health Organization (WHO): sperm concentration < 15×10^6^/mL, and the proportion of forward-moving sperm < 32%; both test results within 2 to 4 weeks must meet the above criteria, and the lower value is included; in addition, the following additional conditions must also be met: (1) No history of reproductive tract infection or inflammation in the past 3 months, and the results of semen bacterial culture, mycoplasma and chlamydia tests are all negative; (2) No organic lesions in the urinary reproductive system; (3) No use of antibiotics or related biological products in the past month; (4) No systemic underlying diseases; (5) No genetic factors such as chromosomal abnormalities.

All participants signed the informed consent form before being included in the study. The research followed the principles of the Helsinki Declaration and was approved by the Ethics Committee of the Affiliated Hospital of Chengdu University of Traditional Chinese Medicine (Ethical Approval Number: 2024KL-016).

### 2.2 Sample collection

All semen samples were collected by the Andrology Laboratory of the Affiliated Hospital of Chengdu University of Traditional Chinese Medicine and were independently evaluated by two trained laboratory technicians using a blinded method. The testers were blinded to the group information. semen was collected through masturbation. After 3 to 5 days of abstinence, it was stored in a sterile glass container. The semen samples were obtained through masturbation under sterile conditions. The samples were stored at -80 °C in a refrigerator within 2 hours after collection. Microbial 16S rRNA gene sequencing. The two collections were spaced 2 to 4 weeks apart.

### 2.3 16S diversity sequencing

#### 2.3.1 DNA extraction and PCR amplification

The genomic DNA of the semen samples was extracted using the DNA extraction kit. Then, the concentration and purity of the DNA were detected by agarose gel electrophoresis and NanoDrop2000. Using the extracted genomic DNA as the template, specific primers with barcodes were used, along with Takara’s Tks Gflex DNA Polymerase, to perform PCR amplification of the bacterial 16S rRNA gene according to the selection of the sequencing region. To ensure the amplification efficiency and accuracy, the upstream primer 343F (5ʹ-ACTCCTACGGGAGGCAGCAG-3ʹ) and the downstream primer 798R (5ʹ- GGACTACHVGGGGTWTCTAAT-3ʹ) were used to PCR amplify the variable region V3-V4 of the gene (Nossa et al., 2010).

#### 2.3.2 PCR amplification and library construction

The PCR amplification products were detected by agarose gel electrophoresis. Then, they were purified using AMPure XP beads. After purification, they were used as templates for a second round of PCR amplification, which was carried out in the same way as the first round. Next, the purified second-round products were subjected to Qubit concentration detection. After the concentration was adjusted, sequencing was performed using the Illumina NovaSeq 6000 sequencing platform, generating 250 bp paired-end reads. The sequencing was supported by Shanghai Ouyi Biotechnology Co., Ltd. (Shanghai, China).

#### 2.3.3 Bioinformatics analysis

The construction of the research library, high-throughput sequencing and basic data analysis were assisted by Shanghai Ouyi Biomedical Technology Co., Ltd. The raw data obtained from the sequencing was in FASTQ format. Before the formal data analysis, the primer fragments in the sequencing sequences were first removed using the Cutadapt software. Then, through the DADA2 process (Callahan et al., 2016) combined with the default parameters of QIIME 2 (2020.11), the quality control filtering, noise reduction, and chimeric removal were performed on the paired-end sequencing data, and finally the characteristic sequences and ASV abundance table were obtained. Subsequently, the characteristic sequences were compared with the Silva 138 database, and species annotation was completed using q2-feature-classifier. Next, the microbiome diversity analysis was carried out using the QIIME 2 software (Bolyen et al., 2019), and the α diversity was evaluated using Chao1 and Shannon indices; Principal coordinate analysis (PCoA) was conducted based on the Binary-Jaccard distance matrix to characterize the β-diversity of the samples. The species abundance differences between groups were compared using the Wilcoxon test (Chao and Bunge, 2002; Hill et al., 2003).

#### 2.3.4 Pollution control and data quality control

To minimize potential contamination and batch effects in low biomass semen samples, the following quality control measures were implemented:(1) The sample processing sequence was randomized, with the case group and control group evenly distributed across each sequencing batch to avoid batch-related confounding; (2) Only samples with a DNA concentration of ≥1.0 ng/μL were used for library construction to ensure sufficient biological material for reliable amplification; (3) The ASVs detected by sequencing were cross-validated with the common reagents/environmental pollutants list published in large-scale low biomass studies (Salter et al., 2014; Glassing et al., 2016) and the Zymo BIOMICS standard product data, and the genera that were consistently reported as laboratory contaminants (such as *Cutibacterium*, *Ralstonia*, *Sphingomonas*, *Methylobacterium*) were manually excluded.

### 2.4 Explainable machine learning model construction and analysis

The differential bacterial communities selected through the Wilcoxon test were used as the characteristic variables. To avoid the inherent compositional effects of the microbiome data, the centered logarithmic ratio (CLR) transformation was applied to standardize the original abundance data. A pseudo-count of 1 was added prior to CLR transformation to accommodate zero values.

In order to determine the core microbiota markers associated with IOA, this study employed three machine learning methods to simultaneously screen for features. The LASSO regression was used, applying L1 penalty to eliminate irrelevant genus features. The LASSO regression was conducted using 10-fold cross-validation (implemented by the “cv.glmnet” function with parameter nfolds=10) to select the optimal λ value by minimizing the binomial bias. The cross-validation was repeated five times to stabilize the results. RF is a tree-based algorithm that ranks the importance of genera based on variable contribution scores. The RF model was implemented using repeated 10-fold cross-validation (five repetitions) with the caret package to assess the importance of variables and reduce the risk of overfitting, selecting the genera with the top 15 importance rankings as candidate features. SVM-RFE identifies key genera by repeatedly eliminating the features with the least information. SVM-RFE integrates 5-fold cross-validation (parameter k=5) into the recursive elimination process using the “rfe” function in the caret package, selecting the feature set with the highest average cross-validation accuracy. The genera features selected by the above three methods were taken as the final core microbiota markers to ensure the robustness and reliability of the feature selection.

To prevent information leakage and provide unbiased performance estimates, we employed a nested, repeated, and stratified cross-validation framework (5-fold × 20 repeats). Within each outer training fold, all preprocessing—including near-zero variance filtering, CLR transformation, and feature selection by three algorithms—was performed strictly on the training data only, with outer test folds used solely for final prediction.

The final classifier was a Firth-penalized logistic regression (brglm2::brglmFit). Model discrimination and calibration were evaluated from participant-level averaged predictions. Genera with a selection frequency≥0.6 across 100 outer models were retained as stable markers.

In this study, the “fastshap” package (with nsim set to 100 for improved stability) was used to calculate the SHAP values for each bacterial genus directly from the final logistic regression model. The distribution of SHAP values was visualized using the “shapviz” package in the form of swarm plots. The contribution value, direction of effect (protection/risk), and abundance-dependent effect of each microbial characteristic on disease classification were quantified at both the global and individual levels. Finally, the core microbial markers of the disease were determined.

### 2.5 Function prediction

To investigate the predicted functional potential of the core microbiota, ASV sequences corresponding to the machine-learning-identified core bacterial genera were analyzed using PICRUSt2 within the QIIME 2 platform. Representative ASV sequences were placed into a reference phylogenetic tree, and microbial gene families were inferred and mapped to KEGG Orthology (KO) terms and pathways. The predicted functions were summarized at KEGG Levels 1-3, and differences between the IOA and FC groups were assessed using the independent-samples Student’s t-test. And a value < 0.05 was considered statistically significant. Because taxon-stratified contribution analysis was not performed, these findings were interpreted as sequence-based predictions of microbial functional potential rather than direct evidence of gene expression, metabolic activity, or the causal mechanisms underlying IOA.

### 2.6 Statistical methods

This study used SPSS^®^ Statistics v22 to analyze clinical and microbiota diversity data. Independent sample t-tests, Wilcoxon rank sum tests, chi-square tests, and Fisher’s exact tests were employed to compare the differences in clinical data, microbiota richness and diversity between the two groups. The differences in microbiota were screened using R language (R version 4.5.3), interpretable machine learning modeling and functional enrichment analysis were conducted. All statistical tests were two-sided, and a *P* value < 0.05 was considered statistically significant.

## 3 Results

### 3.1 Clinical basic information

After rigorous screening and adherence to exclusion criteria, this study included a total of 40 patients with oligoasthenospermia (IOA) and 30 fertile control (FC). **Table 1** summarizes the baseline characteristics of the entire cohort. There were no significant differences between the two groups in terms of age and BMI (*P* > 0.05).

**Table 1:** Basic information for participants.

Characteristics	Healthy group (n=30)	Oligoasthenospermic group (n=40)	p-value
Age(years)	30.60 ± 5.43	32.55 ± 2.71	**0.079**
BMI (kg/m2)	24.93 ± 4.48	24.36 ± 2.71	**0.943**
SMR (%)	65.07 ± 12.07	29.93 ± 8.71	**<0.001**
PR (%)	56.23 ± 10.93	20.72 ± 7.54	**<0.001**
SC(*10^6^/ml)	89.74 ± 49.29	6.10 ± 3.57	**<0.001**
TSC(*10^6^)	263.44 ± 180.70	28.07 ± 20.08	**<0.001**

BMI, body mass index; SMR, Sperm motility rate; PR, Sperm concentration; SC, Sperm concentration; TSC, Total sperm count.

Bold values correspond to P-values from inter-group comparisons. *P* < 0.05 was defined as statistically significant.

### 3.2 Semen microbiome - community state types and microbial diversity

This study obtained high-quality sequences through 16S rRNA gene sequencing of 40 samples from the IOA group and 30 samples from the FC group. Through DADA2 denoising, 11,535 ASVs were obtained. The Ven diagram of ASV levels showed that there were 6,296 unique ASVs in the IOA group and 4,593 unique ASVs in the FC group. The two groups shared 646 ASVs, indicating significant differences in the microbial community composition between the two groups (**Figure 2A**). The dilution curve showed that as the sequencing depth increased, the number of observed species in each group gradually approached a plateau, indicating that the sequencing depth of this study was sufficient to cover the vast majority of microbial species in the samples (**Figure 2B**). The Good’s Coverage curve showed that the coverage of all samples was close to 1.0, indicating that the sequencing results were reliable and could reflect the true composition of the microbial community in the samples (**Figure 2C**). The analysis of species composition at the phylum level showed that *Bacteroidota* and *Firmicutes* were the absolute dominant phyla in the two semen samples, and these two phyla accounted for more than 85% of the total semen microbiota. The proportion of the *Bacteroidota* decreased in the IOA group, while the proportion of the *Firmicutes* increased(**Figure 2D**); at the genus classification level, Muribaculaceae, *Lachnospiraceae_NK4A136_group*, *Lactobacillus*, and *Bacteroides* were the dominant core genera in the semen samples of both groups, and these four genera accounted for more than 50% of the total semen microbiota. Of note, *Prevotella* abundance was significantly elevated in the IOA group compared with the FC group, while other *Bacteroidota* taxa, notably *Bacteroides*, were reduced(**Figure 2E**). This genus-level divergence contributed to the net phylum-level decline of *Bacteroidota*.

**Figure 2:**
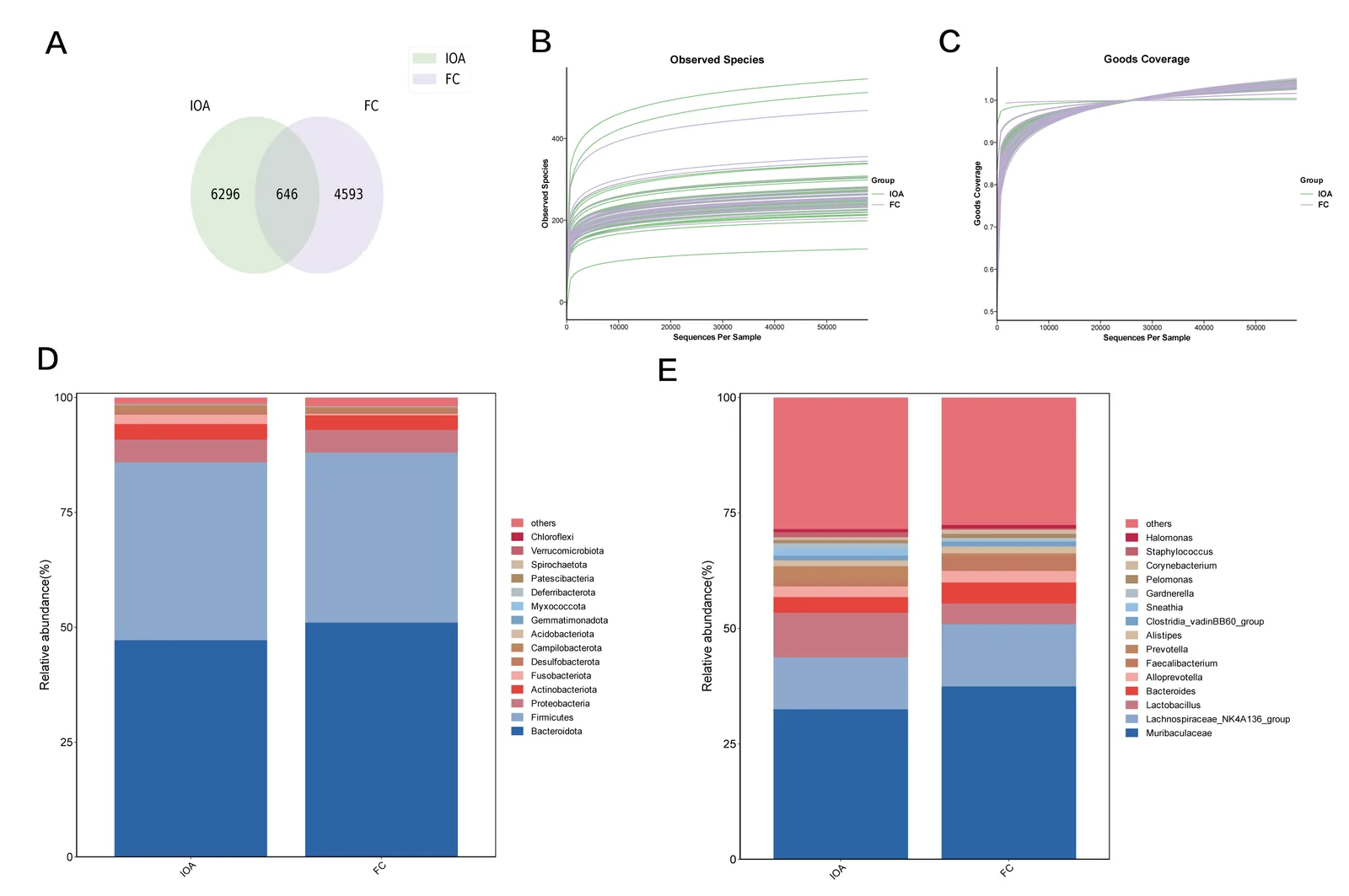
**(A)** Venn diagram of two groups of species. **(B)** Sample dilution curve. **(C)** Good’s Coverage Curve. **(D)** Bar chart showing the relative abundance of microbial communities at the phylum level. **(E)** Bar chart showing the relative abundance of microbial communities at the genus level.

To assess the differences in microbial community diversity between the IOA group and the FC group, this study conducted α diversity and β diversity analyses. The α diversity analysis results showed that the Chao1 index, which reflects species richness (**Figure 3A**), and the Shannon index, which reflects community diversity (**Figure 3B**), showed no significant statistical differences between the two groups. This suggests that the species richness and overall community diversity levels of the two sample groups were similar. The principal coordinate analysis (PCoA) based on Binary-Jaccard distance showed that the microbial community compositions of the IOA group and the FC group exhibited a clear inter-group separation trend. The PERMANOVA test further confirmed that the community structure differences between the two groups were statistically significant (F = 1.08, R²=0.016,*P* = 0.0160) (**Figure 3C**). The PERMDISP test showed no significant difference in multivariate dispersion between groups (F = 0.0027,*P* = 0.963)(**Figure 3D**), confirming that the observed separation reflects a genuine community centroid shift rather than differential group variances.

**Figure 3:**
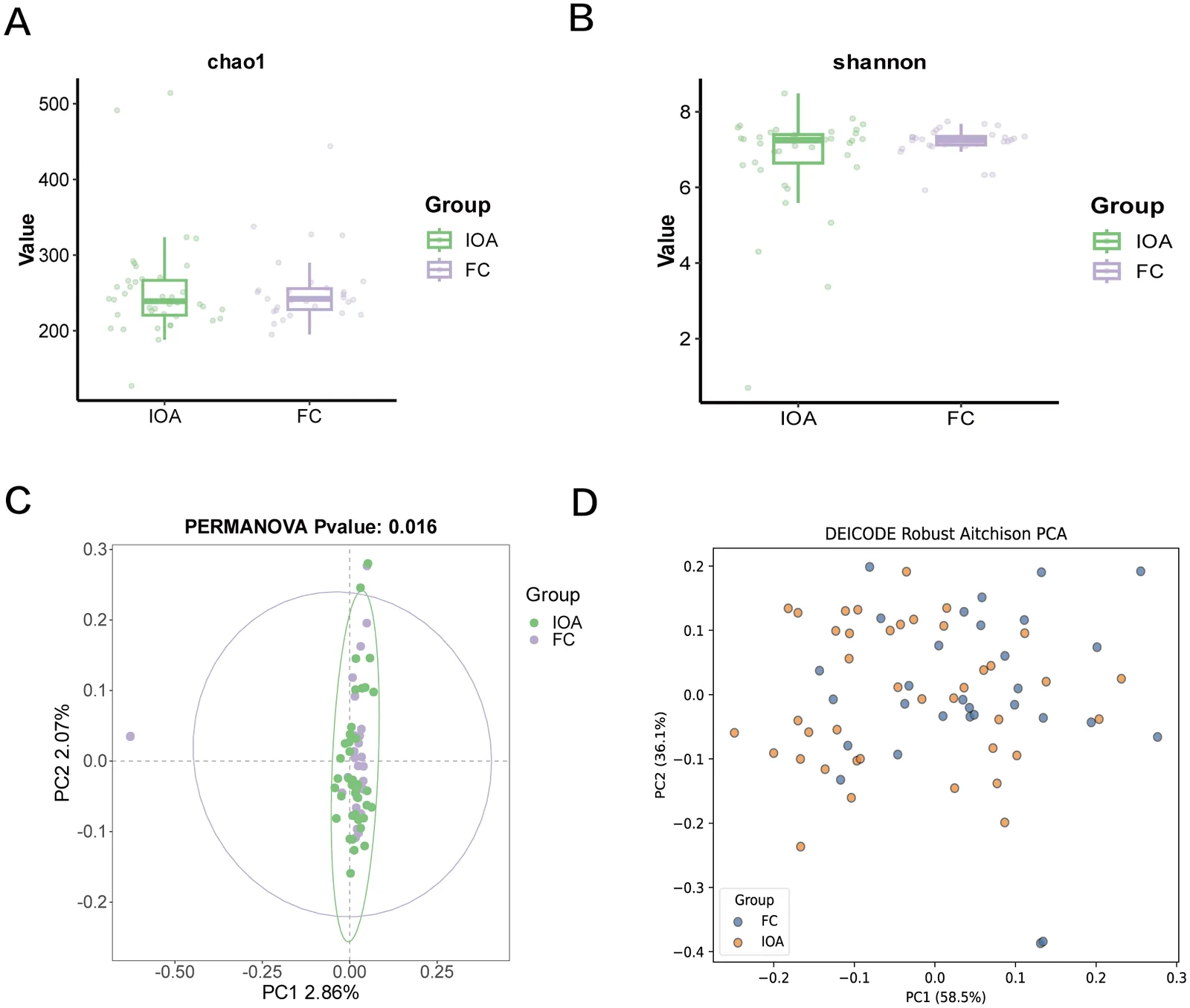
Analysis of biodiversity in biological communities. **(A)** alpha diversity analysis (based on the Chao1 index). **(B)** alpha diversity analysis (based on Shannon index). **(C)** Beta diversity analysis(PcoA plot based on Binary-Jaccard distance). **(D)** Deicode (Robust Aitchison PCA) ordination plot based on Aitchison distance.

### 3.3 Screening of core microbial markers in IOA and construction of diagnostic models

To systematically screen the key microbial groups related to the onset of IOA and construct a non-invasive diagnostic model with clinical application potential, this study adopted a multi-step analysis strategy, combining differential abundance analysis, machine learning feature selection, and model interpretation methods, to conduct in-depth exploration of the semen microbial group data of the IOA group and the FC group.

Firstly, the Wilcoxon rank sum test was used to conduct a difference analysis on the relative abundances of the microbial communities at the genus level between the two groups. A total of 24 significantly different genera were identified (**Supplementary Table 1**). The box plots of the top 10 different genera are shown in **Figure 4A**. These genera, including *Bacteroides*, *Prevotella*, *Nesterenkonia*, and *Blautia*, showed significant abundance distribution differences between the two groups.

**Figure 4:**
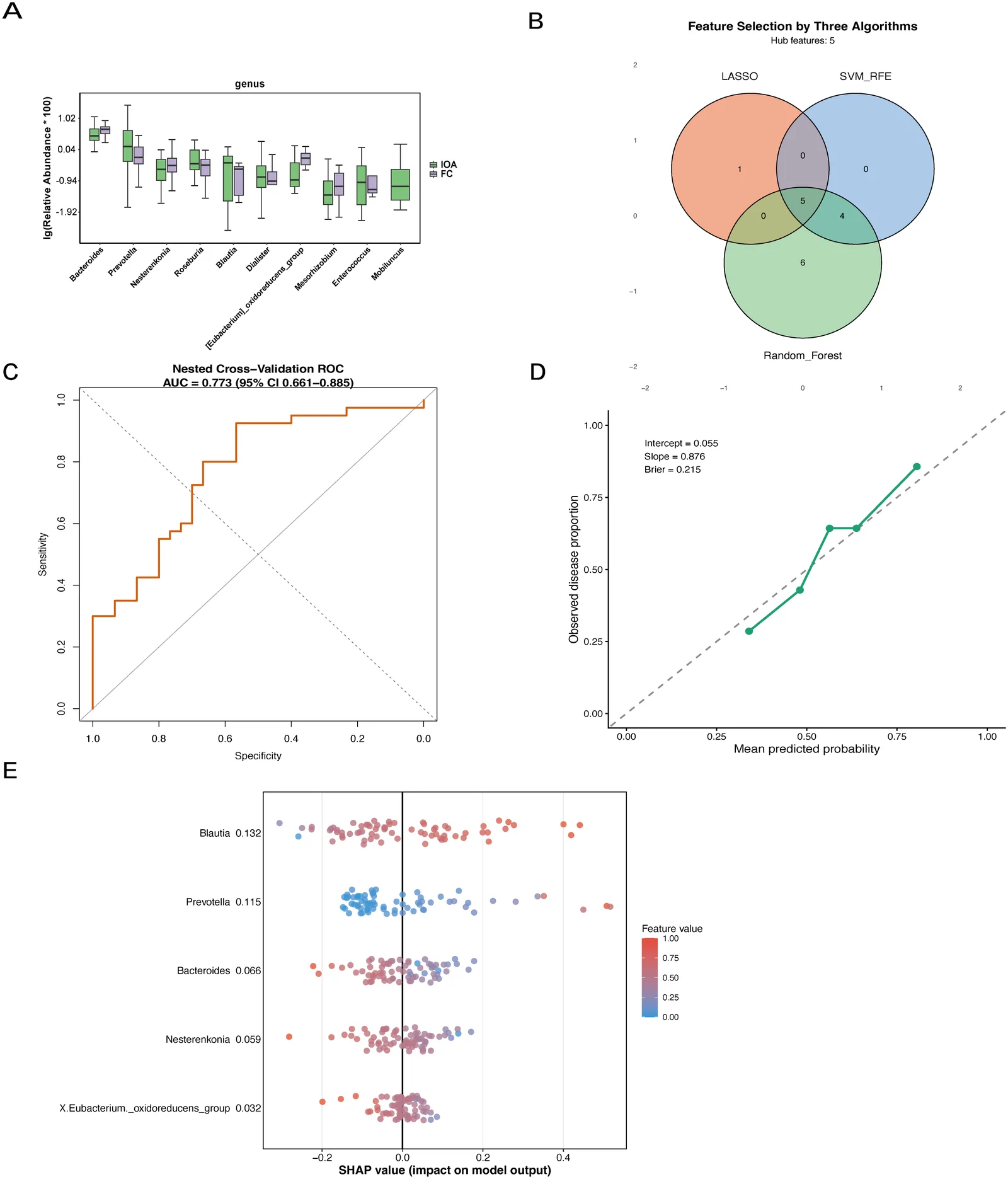
**(A)** Top10 boxplot of the abundance of different species. **(B)** Overlap of Microbial Features Screened by Three Independent Algorithms. **(C)** Receiver Operating Characteristic Curve. **(D)** Calibration plot of the final prediction model. **(E)** SHAP Summary Plot of Core semen microbiota.

To further identify the core microbial characteristics that contribute independently to disease classification, we employed three machine learning algorithms. The RF algorithm identified the top 15 candidate bacterial genera, LASSO regression selected 6 core genera, and SVM-RFE algorithm identified 9 important genera. The intersection of the results from these three algorithms revealed that 5 robust hub microbial genera were simultaneously selected (**Figure 4B**). The Receiver Operating Characteristic (ROC) curve analysis showed that the diagnostic model constructed based on these hub features had good discriminative efficacy, with an area under the curve (AUC) of 0.773 (95% CI: 0.661–0.885) (**Figure 4C**). Additionally, other performance metrics of the model are presented in **Supplementary Table 2**, and the calibration curve demonstrated good calibration ability (**Figure 4D**).

To deeply explain the prediction mechanism of the model and clarify the contribution degree and influence direction of each bacterial genus to disease prediction, this study introduced the SHAP analysis method. The average absolute SHAP value reflects the contribution degree of each bacterial genus to the model prediction, and the sign of the SHAP value reveals the association direction between the abundance of the bacterial genus and disease prediction: a positive value indicates that the higher the abundance of the bacterial genus, the greater the probability that the model predicts IOA; a negative value is the opposite. The color gradient of the bacterial genus points reflects its own abundance level, visually presenting the “abundance - contribution - disease risk” association pattern. As shown in **Figure 4E**, *Blautia* contributed the most to the model prediction (average absolute SHAP = 0.132), followed by *Prevotella* (0.115), *Bacteroides* (0.066), *Nesterenkonia* (0.059), and *[Eubacterium]_oxidoreducens_group* (0.032). Overall, *Blautia* mainly had a negative effect. Most samples with high abundance corresponded to negative SHAP values, suggesting that an increase in its abundance could reduce the risk of IOA prediction, making it a protective bacterial group. *Prevotella* showed a stable positive correlation. The higher the abundance, the more the sample SHAP value tended to be positive, indicating that the enrichment of this bacterium could increase the probability of the sample being classified as IOA. *Bacteroides* exhibited a bidirectional effect feature. Some low-abundance samples had positive SHAP values, while high-abundance samples tended to have negative SHAP values. *Nesterenkonia* and *[Eubacterium]_oxidoreducens_group* had a dominant positive effect overall. An increase in abundance tended to increase the probability of IOA prediction. This result not only verifies the biological rationality of the model but also provides a key target for subsequent research on the microbial mechanism related to IOA.

### 3.4 Metabolic pathways associated with IOA

In order to study the functional changes of the semen microbial community in IOA patients, metabolic pathway enrichment analysis and functional prediction were conducted using the KEGG database to analyze the potential roles of the 5 core bacterial genera selected by machine learning in IOA patients. The KEGG functional prediction showed that the semen microbiota in the IOA group exhibited changes in the predicted functional profile compared to the FC group. In the primary pathways, the predicted functional potential of genetic information processing was significantly enriched in the IOA group (*P* = 1.6×10^-2^) (**Figure 5A**); its 6 subordinate secondary functional modules (nucleotide metabolism, translation, replication and repair, transcription, protein processing and membrane transport) were all significantly enriched (*P* < 0.05) (**Figure 5B**). In the tertiary pathways, glycolysis/gluconeogenesis, purine metabolism, pyrimidine metabolism, ribosome, aminoacyl-tRNA biosynthesis and amino sugar and nucleotide sugar metabolism were the main differentially enriched pathways, among which glycolysis/gluconeogenesis was the most significantly enriched (*P* = 6.67×10^-3^) (**Figure 5C**). The overall enrichment degree of these pathways in the IOA group was higher, suggesting that the semen microbiota of IOA patients showed a deviation in the direction of predicted functions in terms of carbohydrate metabolism, nucleic acid metabolism and genetic information processing.

**Figure 5:**
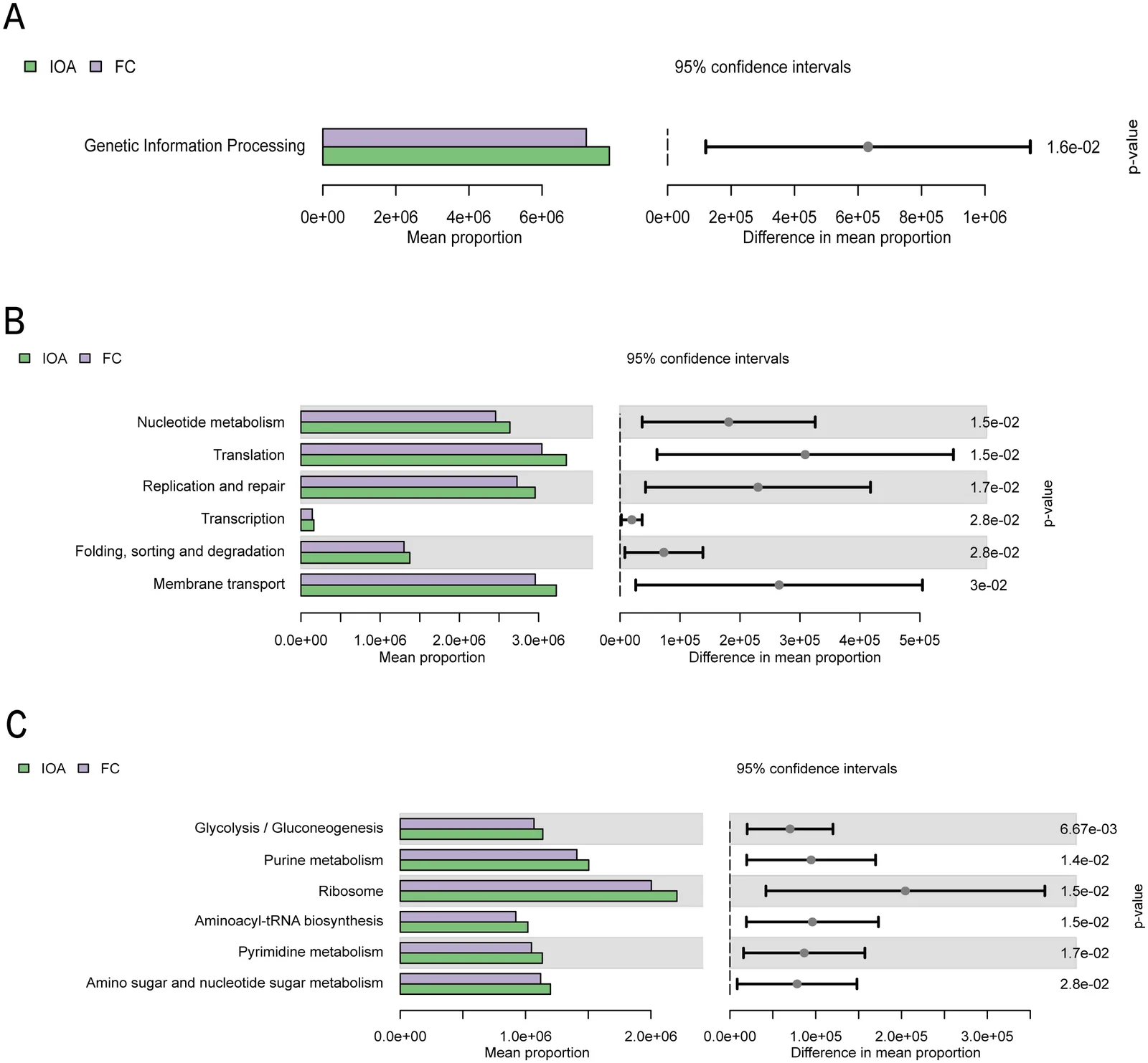
Forest plots showing differential KEGG pathways between the IOA group and FC group. **(A)** KEGG Level 1 pathway inter-group difference forest plot. **(B)** KEGG Level 2 pathway inter-group difference forest plot. **(C)** KEGG Level 3 pathway inter-group difference forest plot.

## 4 Discussion

A balanced microbiome is an integral part of the human body. Advances in next-generation sequencing technology and bioinformatics have shown that human semen is not sterile but rather a dynamic ecosystem containing various microorganisms, which have potential impacts on male fertility and reproductive health (Monteiro et al., 2018; Molina et al., 2025; Weng et al., 2014). Imbalance in the semen microbiome can lead to local inflammation and changes in sperm structure and function (Morawiec et al., 2022). Multiple studies have confirmed that the semen microbiota has the potential to serve as a diagnostic biomarker for male infertility and helps to deeply understand the specific molecular mechanisms of male infertility (Wang et al., 2022; Preetham and Chatterjee, 2025; Yao et al., 2025). Machine learning, as an advanced artificial intelligence algorithm, has been widely applied in screening microbial diagnostic markers for various diseases (Zhang Y et al., 2024; Liu et al., 2023; Chen et al., 2025). Based on this, This study is the first to adopt a multi-algorithm consensus strategy combined with interpretable machine learning methods to systematically characterize the semen microbiota of patients with idiopathic oligoasthenospermia (IOA). We analyzed the bacterial community structure through 16S rRNA sequencing and used three machine learning algorithms in parallel to screen for core bacterial genera, taking the intersection to avoid bias from a single algorithm. At the same time, we combined SHAP analysis to quantify the contribution direction and abundance-dependent effect of each bacterial genus, and finally identified 5 core bacterial genera as potential predictive biomarkers for IOA, and constructed a predictive model with good discrimination performance (AUC = 0.773, 95% CI: 0.661–0.885). Additionally, the functional prediction of the microbiota indicated significant differences in metabolic pathways between the two groups. This method breaks through the limitations of traditional microbiome analysis and provides a new perspective for exploring disease-related microbial biomarkers.

Our research shows that there is no statistical difference between the two groups’ Chao1 index and Shannon index, suggesting that the species richness and overall diversity level of the semen microbiota in the IOA group and the FC group are basically comparable. However, the PCoA analysis based on Binary-Jaccard distance and the PERMANOVA test confirmed that there is a significant differentiation in the species composition and overall structure of the two groups’ microbiota. This result indicates that the semen microecological imbalance related to IOA does not manifest as a large loss or addition of bacterial species, but rather as a reshaping of the internal structure, dominant groups, and relative abundance of species within the original microbiota.

At the phylum level, *Bacteroidota* and *Firmicutes* are the dominant phyla in the semen of the IOA group and the FC group, which was consistent with previous research results (Garcia-Segura et al., 2023; Bazzar et al., 2026; Chen et al., 2018). This suggests that these two phyla may be involved in the regulation of the homeostasis of the semen microenvironment. At the genus classification level, *Prevotella* in the *Bacteroidota* phylum increased in abundance in the IOA group, which was consistent with the trend of community changes at the phylum level. Previous studies have suggested (Grande et al., 2024; Kuribayashi et al., 2026) that excessive proliferation of *Prevotella* may be related to low-grade inflammation in the reproductive tract and increased oxidative stress levels; while the abundance of *Bacteroides* decreased in the IOA group. This alternation in the abundance of these bacterial communities suggests that there may be dysbiosis within the *Bacteroidota* phylum, but whether it is the direct driving factor for the imbalance of the semen microecology in IOA remains to be further verified.

Based on the results of this study and the existing literature evidence, we propose the following working hypotheses. Firstly, *Blautia* has the highest predictive contribution in the SHAP analysis, suggesting that this genus plays a key role in the process of IOA-related semen microecological imbalance. Existing studies have confirmed that *Blautia* is a typical short-chain fatty acid (acetic acid, butyric acid) producer, which can inhibit excessive oxidative stress in the reproductive tract and the activation of the NLRP3 inflammasome, reduce the accumulation of reactive oxygen species (ROS), and alleviate sperm DNA oxidative damage, thereby maintaining the stability of the semen microenvironment (Zhong et al., 2024; Cao et al., 2023). Combined with the SHAP distribution, it can be seen that samples with high *Blautia* abundance mostly correspond to positive protective effects, while samples with low abundance show an increased risk effect, suggesting that it overall behaves as a protective microbiota. Most current related studies focus on the field of intestinal microbiota, and the biological functions and regulatory mechanisms of this bacterium in the semen microenvironment and its regulation still need to be further verified in the future. Secondly, *Bacteroides* is the core genus involved in polysaccharide degradation in the semen microenvironment and can decompose seminal plasma polysaccharides to generate short-chain fatty acids (SCFAs) (Bartsch et al., 2025; Simpson and Campbell et al., 2015; Pan et al., 2026; Huan et al., 2026). SCFAs can serve as substrates for the mitochondrial tricarboxylic acid cycle of sperm mitochondria, improving ATP synthesis efficiency and helping to maintain sperm motility; at the same time, SCFAs can regulate steroid synthesis in interstitial cells and testosterone secretion (Huan P et al., 2026), and inhibit the release of pro-inflammatory factors from the reproductive tract epithelium (Mostafavi Abdolmaleky and Zhou, 2024). The SHAP results of this study show that *Bacteroides* has a significant bidirectional effect: low abundance samples tend to show a risk effect, and with increasing abundance, it gradually shows a protective trend, which is consistent with the potential protective effect reported in the literature. Thirdly, *Prevotella* overgrowth has been reported to be closely related to chronic low-grade inflammation in the reproductive tract (Grande et al., 2024; Kuribayashi et al., 2026). The enrichment of the microbiota can induce inflammatory responses, promote ROS accumulation, and damage sperm motility and DNA integrity (Grande et al., 2024; Baud et al., 2024). The SHAP results of this study show that *Prevotella* has a continuously positive SHAP value as its abundance increases, confirming that the enrichment of this bacterium is a potential risk factor for IOA, which is consistent with previous conclusions. Fourthly, *Nesterenkonia* carries peptidoglycan and other pathogen-related molecular patterns on its cell wall, which can induce local inflammatory responses and affect the stability of the blood-testis barrier (Li et al., 2019; Khani et al., 2025); its genome encodes superoxide dismutase and catalase, enabling it to tolerate the high ROS environment in the semen and continuously release metabolites (Aliyu et al., 2016; Chander et al., 2017). In this study, *Nesterenkonia* mostly shows a positive SHAP effect, supporting its potential promoting effect on the risk of IOA. Finally, the *[Eubacterium]_oxidoreducens_group* as a whole shows a predominantly positive SHAP effect, suggesting that an increase in abundance may increase the probability of IOA, but there are relatively scarce studies related to reproduction, and the specific biological role is still unclear, and further exploration is needed.

This study employed PICRUSt2 for functional prediction, which also revealed the differences in bacterial gene functional potential between the two groups, supporting the view that the semen microbiota may participate in the pathogenesis of IOA by regulating the host’s metabolism and immune response. We identified differential pathways such as glycolysis/gluconeogenesis, purine metabolism, ribosomes, and aminoacyl-tRNA biosynthesis, suggesting that the predicted functions of the IOA-related microbiota in carbohydrate and nucleic acid metabolism are shifted. These findings suggest that microbial metabolic reprogramming may disrupt the homeostasis of the semen microenvironment. We hypothesize that the depletion of protective bacterial genera (such as *Bacteroides*) and the enrichment of *Prevotella* and *Nesterenkonia* may jointly drive the enhancement of the predicted potential for anaerobic glycolysis and membrane transport, promoting the release of pro-inflammatory products and subsequent inflammation and oxidative stress (Chen et al., 2020; Raetz and Whitfield, 2002). In the future, it is urgently necessary to integrate metagenomics, metabolomics, and proteomics to elucidate the mechanisms by which the semen microbiota regulates metabolism, immunity, and signaling pathways, and to provide new targets for the treatment of IOA.

This study acknowledges several limitations. Firstly, it is a single-center cross-sectional study with a limited sample size, which can only confirm the correlation between the microbiota and IOA but cannot establish a causal relationship between them. Secondly, relying solely on 16S rRNA sequencing combined with KEGG functional prediction analysis to determine the metabolic characteristics of the microbiota lacks the use of methods such as metagenomics and *in vitro* cell experiments to verify the true pathogenic mechanisms of the core bacterial genera and differential pathways. Moreover, the microbiota diagnostic model has only undergone internal validation and lacks independent external validation to test its generalization ability, and the clinical translational value still needs to be supported by subsequent experiments. In future studies, large-scale multi-center research must be conducted to verify the stability and universality of the core microbiota characteristics and diagnostic model, and through longitudinal dynamic samples, clarify the causal temporal relationship between the imbalance of key bacterial genera and the onset of IOA. Secondly, by combining multi-omics technologies such as metagenomics and metabolomics, the true functional changes of core bacteria and pathways can be precisely analyzed, and through *in vitro* cell experiments and animal models, the specific molecular mechanisms by which key metabolic pathways disruption mediates sperm damage can be verified. Finally, through external validation and targeted microbiota intervention trials, further exploration of microecological regulatory strategies targeting core bacterial genera can be carried out, providing clinical translational evidence for non-invasive diagnosis and microbial treatment of idiopathic oligoasthenozoospermia.

## 5 Conclusion

In conclusion, this study found that *Blautia*, *Prevotella*, *Bacteroides*, *Nesterenkonia*, and *[Eubacterium]_oxidoreducens_group* have the potential to distinguish patients with IOA from FC, suggesting that they may serve as predictive microbial biomarkers. These microbial differences may be involved in immune dysfunction and metabolic reprogramming, particularly in glycolysis/gluconeogenesis, providing new insights into the dysbiosis of the semen microbiome in IOA. This study emphasizes the potential role of the aforementioned core genera in the pathogenesis of IOA, where *Blautia* acts as a protective factor, while *Prevotella*, *Nesterenkonia*, and *[Eubacterium]_oxidoreducens_group* act as risk factors. We suggest that future studies should prioritize clinical validation of these biomarkers in independent cohorts and integrate them into non-invasive screening protocols. Moving in these directions is expected to improve the early diagnosis of IOA and provide valuable insights into the role of microbial factors in male reproductive health.

## Data Availability

The original data of the microbial group sequencing reported in this article has been stored in the National Genomics Data Center (Nucleic Acid Research 2025), and the Genomic Sequence Archive of the Chinese Academy of Sciences/Biological Information Center/Beijing Genomics Institute (GSA: CRA035998) (Genomics, Proteomics and Bioinformatics 2025), and can be accessed publicly at https://ngdc.cncb.ac.cn/gsa.

## References

[B45] Aliyu H. De Maayer P. Cowan D. (2016). The genome of the Antarctic polyextremophile Nesterenkonia sp. AN1 reveals adaptive strategies for survival under multiple stress conditions. FEMS Microbiol. Ecol. 92, fiw032. doi: 10.1093/femsec/fiw032 26884466

[B37] Bartsch M. Vital M. Woltemate S. Bouwman F. G. Berkemeyer S. B. Hahn A. . (2025). Microbiota-dependent fiber responses: a proof-of-concept study on short-chain fatty acid production in Prevotella- and Bacteroides-dominated healthy individuals. J. Nutr. 155, 3809–3822. doi: 10.1016/j.tjnut.2025.08.034 40914513 PMC12799433

[B42] Baud D. Zuber A. Peric A. Pluchino N. Vulliemoz N. Stojanov M. (2024). Impact of semen microbiota on the composition of seminal plasma. Microbiol. Spectr. 12, e0291123. doi: 10.1128/spectrum.02911-23 38349179 PMC10913749

[B31] Bazzar B. Reshadfar E. Namdar P. Pourbagher D. Bakhtiari Lafmejani R. Soleimanzadeh A. (2026). The relationship between seminal microbiome and male reproductive wellness: a systematic review†. Biol. Reprod. 114, 1151–1167. doi: 10.1093/biolre/ioag003 41615012

[B15] Bolyen E. Rideout J. R. Dillon M. R. Bokulich N. A. Abnet C. C. Al-Ghalith G. A. . (2019). Reproducible, interactive, scalable and extensible microbiome data science using QIIME 2. Nat. Biotechnol. 37, 852–857. doi: 10.1038/s41587-019-0209-9 31341288 PMC7015180

[B14] Callahan B. J. McMurdie P. J. Rosen M. J. Han A. W. Johnson A. J. Holmes S. P. (2016). DADA2: high-resolution sample inference from Illumina amplicon data. Nat. Methods 13, 581–583. doi: 10.1038/nmeth.3869 27214047 PMC4927377

[B35] Cao T. Wang S. Pan Y. Guo F. Wu B. Zhang Y. . (2023). Characterization of the semen, gut, and urine microbiota in patients with different semen abnormalities. Front. Microbiol. 14, 1182320. doi: 10.3389/fmicb.2023.1182320 37293215 PMC10244769

[B46] Chander A. M. Nair R. G. Kaur G. Kochhar R. Dhawan D. K. Bhadada S. K. . (2017). Genome insight and comparative pathogenomic analysis of Nesterenkonia jeotgali strain CD08_7 isolated from duodenal mucosa of celiac disease patient. Front. Microbiol. 8, 129. doi: 10.3389/fmicb.2017.00129 28210247 PMC5288335

[B16] Chao A. Bunge J. (2002). Estimating the number of species in a stochastic abundance model. Biometrics 58, 531–539. doi: 10.1111/j.0006-341x.2002.00531.x 12229987

[B4] Chatzokou D. Tsarna E. Davouti E. Siristatidis C. S. Christopoulou S. Spanakis N. . (2025). Semen microbiome, male infertility, and reproductive health. Int. J. Mol. Sci. 26, 1446. doi: 10.3390/ijms26041446 40003912 PMC11854939

[B11] Chen J. Bai J. Chen B. Huang Y. Li J. Chen Z. . (2026). Oxidative stress reprograms benign prostatic hyperplasia microenvironments: insights from integrative multi-omics and machine learning. J. Transl. Med. 24, 670. doi: 10.1186/s12967-026-08055-8 41923163 PMC13169760

[B3] Chen J. Chen J. Fang Y. Shen Q. Zhao K. Liu C. . (2023). Microbiology and immune mechanisms associated with male infertility. Front. Immunol. 14, 1139450. doi: 10.3389/fimmu.2023.1139450 36895560 PMC9989213

[B29] Chen C. Deng C. Li Y. He S. Liu Y. Pan S. . (2025). Machine learning-derived diagnostic model of epithelial ovarian cancer based on gut microbiome signatures. J. Transl. Med. 23, 319. doi: 10.1186/s12967-025-06339-z 40082895 PMC11905570

[B32] Chen H. Luo T. Chen T. Wang G. (2018). Seminal bacterial composition in patients with obstructive and non-obstructive azoospermia. Exp. Ther. Med. 15, 2884–2890. doi: 10.3892/etm.2018.5778 29456693 PMC5795641

[B47] Chen L. Wen C. W. Deng M. J. Ping-Li Zhang Z. D. Zhou Z. H. . (2020). Metabolic and transcriptional changes in seminal plasma of asthenozoospermia patients. Biomed. Chromatogr. 34, e4769. doi: 10.1002/bmc.4769 31808565

[B7] Davies R. Minhas S. Jayasena C. N. (2023). Next-generation sequencing to elucidate the semen microbiome in male reproductive disorders. Med. (Kaunas) 60, 25. doi: 10.3390/medicina60010025 38256286 PMC10819355

[B6] Farahani L. Tharakan T. Yap T. Ramsay J. W. Jayasena C. N. Minhas S. (2021). The semen microbiome and its impact on sperm function and male fertility: a systematic review and meta-analysis. Andrology 9, 115–144. doi: 10.1111/andr.12886 32794312

[B10] Fei T. Funnell T. Waters N. R. Raj S. S. Baichoo M. Sadeghi K. . (2024). Scalable log-ratio lasso regression for enhanced microbial feature selection with FLORAL. Cell Rep. Methods 4, 100899. doi: 10.1016/j.crmeth.2024.100899 39515336 PMC11705925

[B30] Garcia-Segura S. Del Rey J. Closa L. Garcia-Martínez I. Hobeich C. Castel A. B. . (2023). Characterization of seminal microbiome of infertile idiopathic patients using third-generation sequencing platform. Int. J. Mol. Sci. 24, 7867. doi: 10.3390/ijms24097867 37175573 PMC10178615

[B19] Glassing A. Dowd S. E. Galandiuk S. Davis B. Chiodini R. J. (2016). Inherent bacterial DNA contamination of extraction and sequencing reagents may affect interpretation of microbiota in low bacterial biomass samples. Gut Pathog. 8, 24. doi: 10.1186/s13099-016-0103-7 27239228 PMC4882852

[B33] Grande G. Graziani A. De Toni L. Garolla A. Ferlin A. (2024). Male tract microbiota and male infertility. Cells 13, 1275. doi: 10.3390/cells13151275 39120306 PMC11312145

[B17] Hill T. C. Walsh K. A. Harris J. A. Moffett B. F. (2003). Using ecological diversity measures with bacterial communities. FEMS Microbiol. Ecol. 43, 1–11. doi: 10.1111/j.1574-6941.2003.tb01040.x 19719691

[B40] Huan P. Wang W. Qi Y. Sun J. Zhou R. Liu L. . (2026). Cynomorium songaricum polysaccharide attenuates high-fat diet-induced testicular dysfunction by modulating the gut microbiota. Int. J. Biol. Macromol. 335, 149114. doi: 10.1016/j.ijbiomac.2025.149114 41276052

[B44] Khani N. Shakeri A. H. Houshmandi S. Ziavand M. Abedi-Soleimani R. Hosseinzadeh N. . (2025). The promising biological role of postbiotics in treating human infertility. Probiotics Antimicrob. Proteins 17, 2166–2178. doi: 10.1007/s12602-025-10458-w 39883398

[B36] Kuribayashi S. Naik N. Miller A. W. Lundy S. D. (2026). Micromanagement: how the male reproductive microbiome shapes male fertility. Fertil. Steril 125, 583–595. doi: 10.1016/j.fertnstert.2026.01.019 41611115

[B43] Li K. J. Chen Z. L. Huang Y. Zhang R. Luan X. Q. Lei T. T. . (2019). Dysbiosis of lower respiratory tract microbiome are associated with inflammation and microbial function variety. Respir. Res. 20, 272. doi: 10.1186/s12931-019-1246-0 31796027 PMC6892239

[B28] Liu X. Liu D. Tan C. Feng W. (2023). Gut microbiome-based machine learning for diagnostic prediction of liver fibrosis and cirrhosis: a systematic review and meta-analysis. BMC Med. Inform. Decis. Mak 23, 294. doi: 10.1186/s12911-023-02402-1 38115019 PMC10731850

[B8] Magoutas K. Leathersich S. Hart R. Ireland D. Walls M. Payne M. (2025). Lower semen quality among men in the modern era-is there a role for diet and the microbiome? Microorganisms 13, 147. doi: 10.3390/microorganisms13010147 39858914 PMC11768045

[B21] Molina N. M. Canha-Gouveia A. Leonés-Baños I. Sola-Leyva A. Vargas E. Ruíz-Durán S. . (2025). The complementary seminovaginal microbiome in health and disease. Reprod. Biomed. Online 50, 104707. doi: 10.1016/j.rbmo.2024.104707 40174296

[B20] Monteiro C. Marques P. I. Cavadas B. Damião I. Almeida V. Barros N. . (2018). Characterization of microbiota in male infertility cases uncovers differences in seminal hyperviscosity and oligoasthenoteratozoospermia possibly correlated with increased prevalence of infectious bacteria. Am. J. Reprod. Immunol. 79, e12838. doi: 10.1111/aji.12838 29500854

[B23] Morawiec E. Czerwiński M. Czerwińska A. B. Wiczkowski A. (2022). Semen dysbiosis-just a male problem? Front. Cell. Infect. Microbiol. 12, 815786. doi: 10.3389/fcimb.2022.815786 36176582 PMC9514095

[B41] Mostafavi Abdolmaleky H. Zhou J. R. (2024). Gut microbiota dysbiosis, oxidative stress, inflammation, and epigenetic alterations in metabolic diseases. Antioxidants (Basel) 13, 985. doi: 10.3390/antiox13080985 39199231 PMC11351922

[B13] Nossa C. W. Oberdorf W. E. Yang L. Aas J. A. Paster B. J. Desantis T. Z. . (2010). Design of 16S rRNA gene primers for 454 pyrosequencing of the human foregut microbiome. World J. Gastroenterol. 16, 4135–4144. doi: 10.3748/wjg.v16.i33.4135 20806429 PMC2932916

[B12] Novielli P. Baldi S. Romano D. Magarelli M. Diacono D. Di Bitonto P. . (2025). Personalized colorectal cancer risk assessment through explainable AI and gut microbiome profiling. Gut Microbes 17, 2543124. doi: 10.1080/19490976.2025.2543124 40760681 PMC12326576

[B39] Pan Y. Li B. Liu L. Wang Z. Liu X. (2026). Gut dysbiosis induces the development of asthenozoospermia through butanoate metabolism. Front. Immunol. 17, 1760881. doi: 10.3389/fimmu.2026.1760881 41929479 PMC13038517

[B25] Preetham K. Chatterjee J. (2025). Research trend on the emerging role of the microbiome in idiopathic male infertility. Antonie van Leeuwenhoek 118, 193. doi: 10.1007/s10482-025-02194-6 41241915

[B48] Raetz C. R. Whitfield C. (2002). Lipopolysaccharide endotoxins. Annu. Rev. Biochem. 71, 635–700. doi: 10.1146/annurev.biochem.71.110601.135414 12045108 PMC2569852

[B18] Salter S. J. Cox M. J. Turek E. M. Calus S. T. Cookson W. O. Moffatt M. F. . (2014). Reagent and laboratory contamination can critically impact sequence-based microbiome analyses. BMC Biol. 12, 87. doi: 10.1186/s12915-014-0087-z 25387460 PMC4228153

[B9] Santos-Júnior C. D. Torres M. D. T. Duan Y. Rodríguez Del Río Á. Schmidt T. S. B. . (2024). Discovery of antimicrobial peptides in the global microbiome with machine learning. Cell. 187, 3761–3778.e16. doi: 10.1016/j.cell.2024.05.013 38843834 PMC11666328

[B38] Simpson H. L. Campbell B. J. (2015). Review article: dietary fibre-microbiota interactions. Aliment. Pharmacol. Ther. 42, 158–179. doi: 10.1111/apt.13248 26011307 PMC4949558

[B24] Wang H. Xu A. Gong L. Chen Z. Zhang B. Li X. (2022). The microbiome, an important factor that is easily overlooked in male infertility. Front. Microbiol. 13, 831272. doi: 10.3389/fmicb.2022.831272 35308385 PMC8924671

[B22] Weng S. L. Chiu C. M. Lin F. M. Huang W. C. Liang C. Yang T. . (2014). Bacterial communities in semen from men of infertile couples: metagenomic sequencing reveals relationships of seminal microbiota to semen quality. PloS One 9, e110152. doi: 10.1371/journal.pone.0110152 25340531 PMC4207690

[B1] World Health Organization (2023). Infertility. Available online at: https://www.who.int/news-room/fact-sheets/detail/infertility (Accessed January 27, 2024).

[B26] Yao H. Luo C. Xian Y. Liu B. Yang T. Tian R. . (2025). Integrated profiling of semen microbiota and metabolome reveals dysregulation in primary idiopathic male infertility. J. Transl. Med. 23, 1092. doi: 10.1186/s12967-025-07139-1 41088280 PMC12522667

[B2] Zhang J. Li X. Wang R. Feng X. Wang S. Wang H. . (2024). DNA methylation patterns in patients with asthenospermia and oligoasthenospermia. BMC Genomics 25, 602. doi: 10.1186/s12864-024-10491-z 38886667 PMC11181631

[B27] Zhang Y. Wu H. Xu R. Wang Y. Chen L. Wei C. (2024). Machine learning modeling for the prediction of phosphorus and nitrogen removal efficiency and screening of crucial microorganisms in wastewater treatment plants. Sci. Total Environ. 907, 167730. doi: 10.1016/j.scitotenv.2023.167730 37852495

[B34] Zhong Y. Deng H. Zhao J. Luo G. Li H. (2024). Effects of acetate-producing Blautia wexlerae on oxidative stress and NLRP3 inflammasome in obesity-associated male infertility. Med. Microbiol. Immunol. 213, 11. doi: 10.1007/s00430-024-00796-x 38940844

[B5] Zuber A. Peric A. Pluchino N. Baud D. Stojanov M. (2023). Human male genital tract microbiota. Int. J. Mol. Sci. 24, 6939. doi: 10.3390/ijms24086939 37108103 PMC10139050

