# Supplementary material for: Interpretable machine learning uncovers core seminal microbial signatures in idiopathic oligoasthenospermia
Source: Front Cell Infect Microbiol. 2026 Sep 16;16:1923816. doi: 10.3389/fcimb.2026.1923816 (PMC13623559; doi:10.3389/fcimb.2026.1923816)
Supplement: Supplementary file 1 [file Table2.docx]

Supplementary Table 2:Performance metrics of the prediction model in the test set.

| **Model** | **AUC (95% CI)** | **PR-AUC** | **Accuracy** | **Precision** | **Sensitivity** | **Specificity** |
| --- | --- | --- | --- | --- | --- | --- |
| Logistic regression | 0.773（0.661-0.885） | 0.735 | 0.686 | 0.696 | 0.800 | 0.633 |
